# Serum Calcification Propensity Represents a Good Biomarker of Vascular Calcification: A Systematic Review

**DOI:** 10.3390/toxins14090637

**Published:** 2022-09-15

**Authors:** Maxime Pluquet, Said Kamel, Gabriel Choukroun, Sophie Liabeuf, Solène M. Laville

**Affiliations:** 1 MP3CV Laboratory, EA7517, Jules Verne University of Picardie, F-80000 Amiens, France; 2 Department of Biochemistry, Amiens University Medical Center, F-80000 Amiens, France; 3 Department of Nephrology, Amiens University Medical Center, F-80000 Amiens, France; 4 Pharmacoepidemiology Unit, Department of Clinical Pharmacology, Amiens University Medical Center, F-80000 Amiens, France

**Keywords:** calcification, T50, serum calcification propensity test, chronic kidney disease

## Abstract

Vascular calcification contributes to cardiovascular morbidity and mortality. A recently developed serum calcification propensity assay is based on the half-transformation time (T50) from primary calciprotein particles (CPPs) to secondary CPPs, reflecting the serum’s endogenous capacity to prevent calcium phosphate precipitation. We sought to identify and review the results of all published studies since the development of the T50-test by Pasch et al. in 2012 (whether performed in vitro, in animals or in the clinic) of serum calcification propensity. To this end, we searched PubMed, Elsevier EMBASE, the Cochrane Library and Google Scholar databases from 2012 onwards. At the end of the selection process, 57 studies were analyzed with regard to the study design, sample size, characteristics of the study population, the intervention and the main results concerning T50. In patients with primary aldosteronism, T50 is associated with the extent of vascular calcification in the abdominal aorta. In chronic kidney disease (CKD), T50 is associated with the severity and progression of coronary artery calcification. T50 is also associated with cardiovascular events and all-cause mortality in CKD patients, patients on dialysis and kidney transplant recipients and with cardiovascular mortality in patients on dialysis, kidney transplant recipients, patients with ischemic heart failure and reduced ejection fraction, and in the general population. Switching from acetate-acidified dialysate to citrate-acidified dialysate led to a longer T50, as did a higher dialysate magnesium concentration. Oral administration of magnesium (in CKD patients), phosphate binders, etelcalcetide and spironolactone (in hemodialysis patients) was associated with a lower serum calcification propensity. Serum calcification propensity is an overall marker of calcification associated with hard outcomes but is currently used in research projects only. This assay might be a valuable tool for screening serum calcification propensity in at-risk populations (such as CKD patients and hemodialyzed patients) and, in particular, for monitoring changes over time in T50.

## 1. Introduction

Cardiovascular complications are among the leading causes of mortality worldwide and are strongly associated with vascular calcification and atherosclerosis. Vascular calcification is frequent in the general population, and its incidence increases with age. The condition contributes to cardiovascular morbidity and mortality in elderly patients, patients with chronic kidney disease (CKD), and patients with diabetes mellitus [1]. Conversely, both diabetes and CKD aggravate the severity and progression of vascular calcification. Vascular calcification is a cell-mediated process that is driven by the dysfunction death and vascular smooth muscle cells (VSMCs) [2]. It results from both passive and active deposition of calcium phosphate in the arterial wall. Under physiological conditions, inhibitors of active mineralization (including matrix Gla protein (MGP), pyrophosphate (PPi), fetuin-A, osteoprotegerin (OPG), adenosine, bone morphogenetic protein 7 (BMP-7) and osteopontin (OPN)) protect blood vessels from the formation of stable hydroxyapatite crystals.

To date, various techniques have been used to detect and quantify vascular calcification. Clinical examinations are mainly based on non-invasive and invasive imaging methods; for example, computed tomography (CT) is one of those most commonly used to analyze vascular calcification. However, the high cost and the exposure to ionizing radiation limit the use of CT in routine clinical practice and in large epidemiologic studies. Furthermore, CT cannot quantify the likelihood of calcification in the future. Various biomarkers have been identified as potential therapeutic targets or predictors of cardiovascular outcomes [3]. However, these biomarkers present several shortcomings. Firstly, they are mainly used in research, rather than in routine clinical practice. Secondly, they correspond to a single, specific calcification pathway and do not track the overall propensity for calcification.

**Figure 1:**
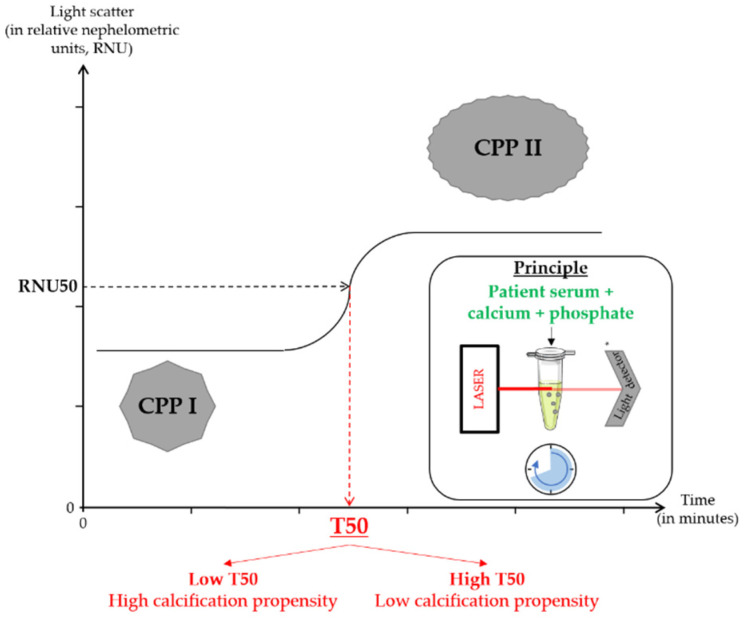
Schematic illustration of the principles underlying the T50 test. * Nephelometer. CPP I: primary calciprotein particles; CPP II: secondary calciprotein particles; RNU: relative nephelometric units; T50: half-transformation time. Note: Primary calciprotein particles (CPP I) spontaneously turn into secondary calciprotein particles (CPP II) when supraphysiological amounts of calcium and phosphate ions are added to serum. CPP I and CPP II differ in how they scatter laser light. Hence, time-resolved nephelometry can be used to detect changes over time in turbidity and thus the transformation of CPP I into CPP II. The T50 corresponds to the time at which 50% of the change in relative nephelometric units are observed; the longer the T50, the slower the transformation of CPP I into CPP II and the lower the serum calcification propensity. Adapted from Pasch et al. [4].

The serum calcification propensity test (also known as the T50 test) has recently been developed as a functional assay of the ability of an individual’s serum to resist apatite crystal formation when supraphysiological amounts of calcium and phosphate ions are added. The biological and physical principles behind the test have been reviewed recently [4]. Under physiological circumstances, the precipitation of supersaturated calcium and phosphate in serum is prevented by the formation of primary calciprotein particles (CPPs), which may subsequently transform to more harmful secondary CPPs. The primary-to-secondary CPP transformation time (also known as the serum T50) reflects the serum’s endogenous ability to prevent calcium phosphate precipitation (Figure 1).

Here, we systematically review scientific publications on serum calcification propensity as studied in vitro, in animal models, or in the clinic.

## 2. Results

**Figure 2:**
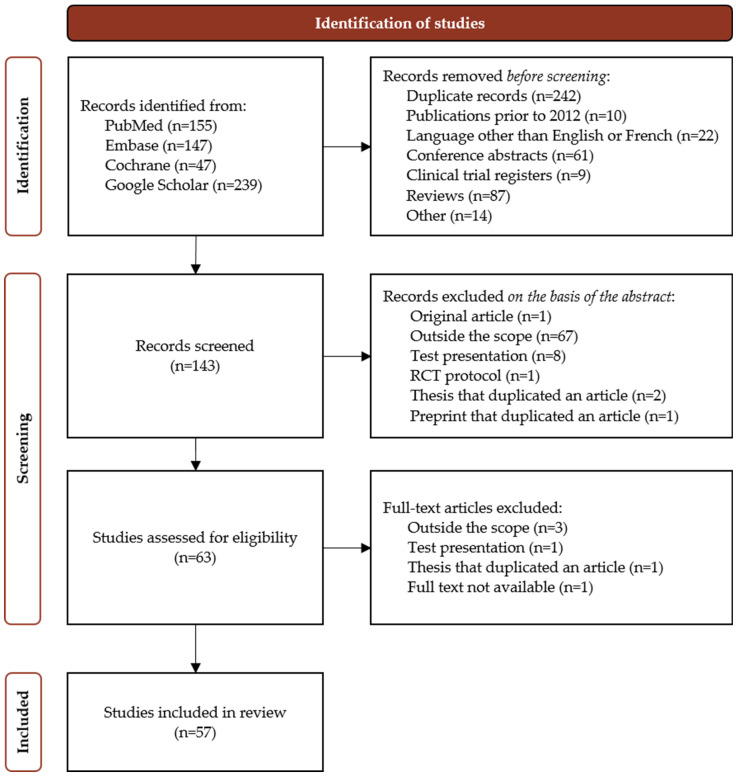
Flow diagram of the selection process.

The PubMed search initially yielded 195 publications; after the elimination of duplicates, 155 publications were selected for further analysis. The query for the Elsevier EMBASE database yielded 147 records, 71 of which were selected after duplicates with PubMed had been removed. The search in the Cochrane Library resulted in 71 hits of which 10 were selected after the removal of publications already identified. The search in Google Scholar yielded 239 publications of which 129 were duplicates. In all, our search of four databases yielded 346 publications after the removal of duplicates (Figure 2).

After this initial selection, 143 records were eligible for further screening (Figure 2). A total of 63 publications were deemed eligible for examination of the full-text reading by at least one of the reviewers. After a further consensus step, 57 publications were included in the review (Figure 2).

The characteristics and outcomes of the reviewed in vitro, animal and clinical studies of T50 are summarized in Table 1 and Table 2. Most of the studies used Pasch et al.’s method to evaluate the T50 [4].

### 2.1. In Vitro Studies

There were two in vitro studies of serum calcification propensity. Firstly, Ter Braake et al. studied the ex vivo influence of Mg^2+^ on calcification propensity in the serum of CKD patients and healthy controls. The researchers found that the addition of 0.2 mmol/L Mg^2+^ significantly lengthened T50 in kidney transplant recipients and healthy controls. Each 0.2 mmol/L increment in the Mg^2+^ concentration was associated with similar increases in kidney transplant recipients and controls; hence, Mg^2+^ lengthened the T50 in a dose-dependent manner [5]. In another study, addition of a physiological concentration of exogenous zinc chloride (ZnCl_2_) significantly lengthened the serum T50 (decreasing the calcification propensity) in samples from hemodialysis patients and healthy volunteers [6].

### 2.2. Animal Studies

There were five animal studies of serum calcification propensity. One of the studies used whole-body Memo1 conditional knock-out (cKO) mice, which displayed premature aging and disturbed mineral metabolism (but not soft-tissue calcification) and a lower serum calcification propensity. T50 and magnesium concentrations were significantly higher in serum samples from Memo1 cKO mice than in control samples. Additional experiments suggested that mice are protected from ectopic calcification by the higher serum magnesium levels resulting from greater expression of magnesium-transporting molecules in the kidneys and the gut [10]. Mutations in the gene coding for platelet-derived growth factor B (PDGFB) in humans are associated with primary familial brain calcification, and mice that are hypomorphic for PDGFB (Pdgfb^ret/ret^) presented brain vessel calcification mimicking the disease observed in human mutation carriers. There was no significant difference in serum T50 values between Pdgfb^ret/ret^ mice and controls [8]. The fact that T50 was significantly greater in serum from Klotho^−/−^ mice than in serum from Klotho^+/−^ or wild-type mice indicated the presence of an impairment in serum calcification buffering capacity in the KO mice [7]. In rats fed a high-adenine, high-phosphate diet, a series of inositol phosphate derivates were examined for their potential to inhibit vascular calcification. At doses of 5, 15, or 50 mg/kg/day, the inositol phosphate analog (OEG2)2-IP4 had no effect on T50 [9]. In a study of rats treated with vitamin D3 to induce vascular calcification, the respective impacts on T50 of intravenous etidronate and FYB-931 (a novel bisphosphonate compound) administered thrice weekly for 2 weeks was compared. T50 was prolonged in a dose-dependent manner by FYB-931 but not by etidronate [11].

### 2.3. Clinical Studies

#### 2.3.1. Observational Studies

##### Chronic Kidney Disease

**Figure 3:**
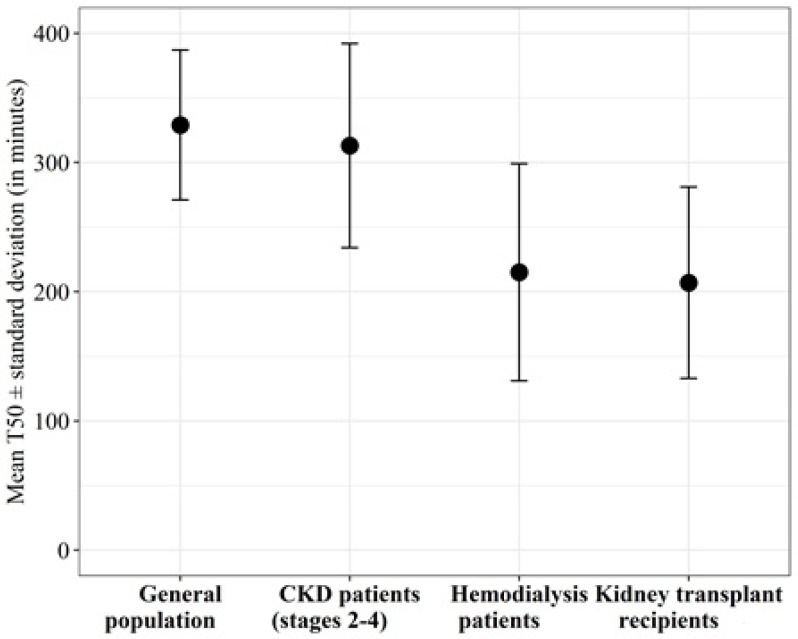
The mean and standard deviation T50 values in four clinical studies [18,30,32,35]. CKD: chronic kidney disease.

We identified nine observational studies of CKD patients not on dialysis. Although T50 is lower in CKD patients than in healthy controls (Figure 3) [19,23,26], Chen et al. did not find a difference in T50 between CKD patients with vascular calcification and those without [26]. T50 is linked to biochemical parameters; it is longer when levels of magnesium, fetuin-A, albumin, and bicarbonate are high and shorter when levels of phosphate and beta cross-laps are high. It is not related to the estimated glomerular filtration rate (eGFR) [21]. T50 is also significantly correlated with serum zinc and uromodulin levels and inversely correlated with serum C-reactive protein (CRP) levels [23,29,37]. It has also been shown that serum calcification propensity is associated with progressive aortic stiffening, higher renal vascular resistance and stiffness, and lower renal tissue oxygenation and perfusion [12,19]. Despite the absence of an association with the prevalence and incidence of coronary artery calcification (CAC), T50 is associated with CAC severity and progression [28]. Smith et al. showed that serum calcification propensity is independently associated with all-cause mortality [12]. The same association was found in Bundy et al.’s study of a cohort of 3404 CKD patients, together with associations with cardiovascular events and end-stage kidney disease; however, these associations were no longer significant after adjustment for kidney function [30].

##### Dialysis

Nine observational studies involved patients on dialysis. First, the initiation and maintenance of hemodialysis or peritoneal dialysis is associated with a lower serum calcification propensity (Figure 3) [16,33]. Compared with healthy controls, patients on dialysis have a lower T50 (Figure 3) [23,24]. In children on hemodialysis, a longer T50 is associated with higher serum levels of magnesium, calcium, and fetuin-A, and a shorter T50 is associated with a higher serum phosphate level [36]. Chen et al. stated that baseline T50 was not associated with pulse wave velocity, the presence and severity of CAC or thoracic artery calcification (TAC), and mortality; however, the size of secondary CPP was associated with all these outcomes, and the size of CPP aggregates is negatively correlated with T50 [38]. Lorenz et al. found that the rate of decline in T50 was a significant predictor of all-cause and cardiovascular mortality, while the cross-sectional T50 values at inclusion and at 24 months were not [20]. In a cohort of 2785 hemodialysis patients, a lower T50 was associated with all-cause mortality, myocardial infarction, and peripheral vascular events [18].

##### Kidney Transplant Recipients

We identified five observational studies of kidney transplant recipients. In a study by Thorsen et al., T50 was significantly higher in a vitamin D-sufficient group than in patients with vitamin D deficiency and insufficiency [32]. T50 decreased with the arterial lesion burden and was inversely associated with more severe interstitial fibrosis [17]. A lower T50 was associated with a greater risk of cardiovascular disease outcomes [25] and graft failure [14,32]. In Dahle et al.’s study of a cohort of 1435 kidney transplant patients, a low serum T50 was associated with increased risks of cardiovascular, cardiac, and all-cause mortality [14,15].

##### Diabetes

Four observational studies have been conducted in diabetic patients: two in type 1 diabetes mellitus (T1DM) and two in type 2 diabetes mellitus (T2DM). T50 was higher in patients with intraperitoneal insulin administration than patients with subcutaneous administration [34]. Calcification propensity was associated with serum marker of mineral stress in T1DM patients [31], and T50 is negatively and independently associated with HbA1c and positively associated with the serum zinc level in T2DM patients [6,39], T50 was not associated with previous macrovascular events and the presence of microvascular disease in T2DM or the development of long-term macrovascular complications in T1DM [31,39].

##### Other

Eight observational studies involved other patient populations. In a genome-wide association study of the general population, three single nucleotide polymorphisms in the *AHSG* gene encoding fetuin-A (rs4917, rs2077119, rs9870756) were identified and together explained 18.3% of the variance in the T50 [41]. In hypertensive patients with preserved kidney function, calcification propensity was higher than in healthy controls [19]. In the general population, T50 was inversely associated with phosphate levels, age, the eGFR, and alcohol consumption, and was positively associated with plasma magnesium levels [35]. In a cohort of patients suffering from primary aldosteronism or resistant hypertension, the decline in T50 over the follow-up period was found to be associated with higher calcium levels, an increase in phosphate levels and a decrease in magnesium levels [42]. In a cohort of men aged 65 or older, there was no significant associations between T50 and total hip or spine bone mineral density [27]. T50 was found to be negatively associated with systemic lupus erythematosus disease activity [22]. In a cohort of 21 living kidney donors, calcification propensity was no worse 1 year after donation [13]. In hypertensive patients, a lower T50 was associated with lower renal tissue oxygenation and perfusion, and higher renal vascular resistance and stiffness [19]. A lower T50 was also associated with a higher atherosclerotic cardiovascular disease score in patients with resistant hypertension and primary aldosteronism. In the latter population, a higher aldosterone-to-renin ratio and greater vascular calcification within the abdominal aorta were associated with a lower T50 [42]. Lastly, serum calcification propensity was associated with an increased risk of cardiovascular mortality in patients with chronic ischemic heart failure and reduced ejection fraction in the general population [35,40].

#### 2.3.2. Interventional studies

##### Dialysis

Three interventional studies were conducted in hemodialysis patients by modulating the composition of the dialysate. Both Lorenz et al. and Ter Meulen et al. compared citrate-acidified vs. acetate-acidified dialysate solutions. In a small, randomized, 4-week crossover study of 18 patients, Ter Meulen et al. found that dialysis with a citrate-acidified solution was associated with a significantly greater T50, relative to an acetate-acidified solution [54]. On the same lines, Lorenz et al. found that 3 months on acetate-free, citrate-acidified dialysis solution led to a higher T50, compared with dialysis with acetate-acidified solution [46]. In a randomized clinical trial (RCT), a high (2.0 mEq/L) magnesium concentration in the dialysate was associated with a significantly greater T50, relative to a standard (1.0 mEq/L) magnesium concentration. During the follow-up period with a switch from high magnesium dialysate to standard magnesium dialysate, T50 returned to baseline levels [48].

##### Phosphate Binders

Four interventional studies of dialysis patients sought to determine the effect of phosphate binders on serum calcification propensity. In patients on peritoneal dialysis, a combination of sevelamer (used as a second-line, low-dose therapy (400 mg 3x/day)) and calcium carbonate was compared with first-line, high-dose sevelamer (800–1200 mg 3x/day). After 2 years of treatment, there were no significant within-group or between-group differences in serum T50 [60]. In an RCT comparing three groups of hemodialysis patients, respectively, taking calcium carbonate alone, sevelamer hydrochloride alone, and sevelamer carbonate alone, T50 increased from baseline over 24 weeks (and to a similar extent) in both the calcium carbonate and sevelamer-treated groups [55]. In a crossover RCT in which patients took sucroferric oxyhydroxide (SO) at a low dose (250 mg/day) or a high dose (2000 mg/day) for 2 weeks and then (after a 2-week washout period) crossed over to the other dose level, only the high-dose treatment was associated with a significantly longer T50 [56]. Lastly, in a 12-week RCT involving 722 hemodialysis patients with refractory hyperphosphatemia, a combination of modified-release nicotinamide, and oral phosphate binder led to a longer T50 than a combination of placebo and phosphate binder [57].

##### Magnesium

Two interventional studies evaluated the effect of magnesium on serum calcification propensity in CKD patients. In a randomized crossover study of patients with stage 3 CKD and CKD patients on dialyses, calcium magnesium citrate was compared with calcium acetate, with a washout period of 1 week between the two treatment phases. In stage 3 CKD patients, neither treatment altered T50. However, in patients on dialysis, calcium magnesium citrate (but not calcium acetate) significantly increased T50 [52]. In the second study, magnesium supplementation (Mg hydroxide, 360 or 720 mg/day) was compared with placebo in CKD stage 3–4 patients. T50 increased significantly in the high-dose group at both weeks 4 and 8 but increased at week 4 only in the low-dosage group. There were no significant changes in the placebo group [44].

##### Other Treatments

There were ten interventional studies with other types of treatment than those listed above. Firstly, in a controlled study of 240 min, CKD patients and healthy controls ingested a standardized meal containing 300 mg calcium and 188 mg phosphate. There were no between-group pairwise differences in T50 [61]. In Mohammad et al.’s comparative study of a high-phosphate diet and a low-phosphate diet in young healthy adults, modulation of the dietary phosphate load for 11 weeks did not significantly affect T50 [47]. Calcium carbonate is frequently used for bone protection in postmenopausal women. Bristow et al. conducted a placebo-controlled RCT of 3 months to check the effect of calcium carbonate on calcification propensity. T50 declined in both groups, and the change was slightly (but not significantly) greater in the calcium group [43]. Three randomized trials have been performed on CKD stage 3 patients and/or CKD stage 4 patients. Allopurinol’s efficacy in improving T50 was tested in hyperuricemic stage 3 CKD patients; after 12 weeks, it had no effect vs. placebo on T50 [51]. The effect of regulating metabolic acidosis in CKD stage 3–4 patients with oral NaHCO_3_ supplementation on calcification propensity was examined in two studies. Firstly, Kendrick et al.’s randomized crossover study compared 6 weeks of treatment with NaHCO_3_ vs. placebo with a 2-week washout period. Secondly, Aigner et al.’s RCT compared high-dose oral NaHCO_3_ supplementation with NaHCO_3_ rescue therapy (though only if necessary) for 4 weeks. Neither trial evidenced an effect of oral NaHCO_3_ supplementation on T50 [50,53]. In an RCT, the synthetic vitamin D analog paricalcitol had no effect on T50 (relative to placebo) during the first year post-transplantation [49]. A post-hoc analysis of an RCT looked at the impact of the bisphosphonate ibandronate on T50 in kidney transplant recipients. T50 increased from baseline to 10 weeks after transplantation and did not change further after 1 year; however, these changes were similar in the placebo and ibandronate groups [45]. Two studies reported on compounds that reduced calcification propensity. Firstly, in a study of hemodialysis patients, T50 was slightly longer in a spironolactone group than in a placebo group [59]. Secondly, a 1-year RCT conducted by Shoji et al. compared the calcimimetic etelcalcetide with the vitamin D receptor activator maxacalcitol in a cohort of 321 hemodialysis patients with secondary hyperparathyroidism. The increase in T50 was significantly greater in the etelcalcetide group than in the maxacalcitol group [58].

## 3. Discussion

To the best of our knowledge, the present systematic review is the first to have examined serum calcification propensity as measured in the T50 test. We believe that our review is exhaustive because we included all the published preclinical studies (in vitro and in animals) and clinical studies (with observational and interventional designs) on T50. Several lines of evidence indicate that T50 is abnormally short in CKD patients in general and in those with end-stage kidney disease (treated with dialysis or transplantation) in particular (Figure 3). A lower T50 was found to be associated with (i) the extent of vascular calcification within the abdominal aorta in patients with primary aldosteronism, and (ii) CAC severity and progression in patients with CKD [19,38]. T50 was also associated with hard outcomes, such as a higher risk of cardiovascular events and all-cause mortality in CKD patients, dialysis patients and kidney transplant recipients [18,20,26,27,30,31,32], and cardiovascular mortality in dialysis patients, kidney transplant recipients, patients with chronic heart failure with a reduced ejection fraction, and in the general population [26,31,32,33,42]. Some therapeutic interventions were found to decrease serum calcification propensity. Changing from acetate-acidified dialysate to citrate-acidified dialysate increased T50 [43,44], as did increasing the dialysate magnesium concentration [45]. Oral magnesium supplementation also extended T50, with effects ween for calcium magnesium citrate in CKD stage 5 [50], and magnesium hydroxide in CKD stages 3–4; this is logical, since magnesium is a calcification inhibitor [51]. Hyperphosphatemia is one of the main factors associated with a high calcification propensity. A few phosphate binders decreased calcification propensity in hemodialysis patients: sevelamer, calcium carbonate, sucroferric oxyhydroxide and modified-release nicotinamide combined with an oral phosphate binder [47,48,49]. The calcimimetic etelcalcetide was associated with a longer T50 in hemodialysis patients with secondary hyperparathyroidism [61], and spironolactone was associated with a slightly longer T50 in hemodialysis patients [60]. It is well known that patients with CKD have a significantly higher risk of vascular calcification and the associated cardiovascular mortality. Hence, the identification of a new biomarker that tracks the overall calcification propensity (when other biomarkers focus on a specific vascular calcification pathway) is of major interest. Indeed, T50 might be valuable for managing vascular calcification in patients with CKD and in other populations prone to developing vascular calcification. Nevertheless, one must take account of the T50 test’s current limitations: it is not routinely available and has to be evaluated centrally by a laboratory in Switzerland, which limits its use to research purposes.

The studies reviewed here are difficult to compare because of differences in their designs, objectives, study populations, durations, and sample sizes. Only 6 of 19 the interventional trials were double-blind, placebo-controlled RCTs, i.e., the gold standard for evaluating a treatment’s efficacy. Our review process was subject to some limitations. Firstly, only one investigator read the full texts. However, a second investigator checked all the abstracts and (in the event of doubt) the corresponding full-text publication. Secondly, we limited our selection to publications in English and French. Nevertheless, we believe that neither of these methodological limitations greatly influence the review’s overall conclusions.

Although serum calcification propensity is an overall calcification marker associated with hard outcomes (such as mortality) in many populations, it is currently applied in research projects only. It would probably be of value to use this tool to evaluate serum calcification propensity in at-risk populations (such as CKD patients and hemodialyzed patients) and especially to monitor changes over time in T50. Furthermore, T50 might become a standard tool for the assessment of vascular calcification and a valuable biomarker in clinical trials designed to identify drugs that slow the progression of vascular calcification. Using T50 as an intermediate endpoint (despite the inherent limitations) might help to shorten trial timelines.

## 4. Methodology

This systematic review was registered in the PROSPERO database (CRD42022355466) and was reported in accordance with the Preferred Reporting Items for Systematic Reviews and Meta-Analyses (PRISMA) guideline.

### 4.1. Search Strategy

To identify studies of serum calcification propensity (the T50 test), we searched the following electronic databases on 11 May 2022: PubMed, Elsevier EMBASE, the Cochrane Library, and Google Scholar. These databases were accessed through the library at the Jules Verne University of Picardie (Amiens, France).

In PubMed, we used the search term “serum calcification propensity” and also applied the “cited by” function (to obtain articles citing Pasch et al.’s original article on the principles of the T50 test) [4].

For the Elsevier EMBASE database, we used the following query: “serum propensity calcification” OR ((“serum”/exp OR serum) AND propensity AND (“calcification”/exp OR calcification)). 

In the Cochrane Library, we searched for “serum calcification propensity” in the “Title-Abstract-Keyword” research field. 

Lastly, in Google Scholar, we used the “cited by” function to obtain articles citing Pasch et al.’s original article [4].

### 4.2. Selection, Screening and Inclusion

We excluded records published before the development of the T50-test in 2012, publications in languages other than English or French, abstracts from congresses or clinical trial registers, reviews, case reports, commentaries, and editorials. Two reviewers independently analyzed the abstracts of the remaining records and determined whether examination of the full text would be required. Disagreements were resolved by discussion and consensus, although a third reviewer could be consulted if necessary. After all eligible full-text publications had been read, we extracted details on the study design, sample size, the setting, characteristics of the study population, the intervention (if any) and the main results on serum calcification propensity (Table 1).

## Figures and Tables

**Table 1:** Summary of in vitro, animal studies about serum calcification propensity using the T50 test.

Study	Experimental Models	Main Results
Ter Braake et al., 2020 [5] Calciprotein particle inhibition explains magnesium-mediated protection against vascular calcification	Human serum with or without the addition of MgCl_2_-containing solutions, resulting in elevations Mg^2+^ concentrations (0.2, 0.4, 0.6, 0.8 and 1.0 mmol/L): Kidney transplant recipients (n = 10) Healthy controls (n = 10)	Mg^2+^ dose-dependently delayed the maturation of CPP1 to CPP2 in vitro. In human serum, addition of 0.2 mmol/L Mg^2+^ significantly lengthened T50 in healthy controls and CKD patients. Each 0.2 mmol/L increment in Mg^2+^ led to a similar increase in T50 in CKD patients and in healthy controls.
Nakatani et al., 2020 [6] Association between Serum zinc and calcification propensity (T(50)) in patients with type 2 diabetes mellitus and in vitro effect of exogenous zinc on T(50)	Pooled serum samples: Healthy volunteers Patients on hemodialysis	Addition of a physiological concentration of exogenous zinc chloride (ZnCl_2_) was shown to significantly increase T50 (and thus decrease the calcification propensity) in serum from healthy volunteers and from patients on hemodialysis.
Mencke et al., 2018 [7] Imaging of incipient vascular calcification in Klotho deficiency	Mice: Klotho^−/−^ (n = 9) Klotho^+/−^ (n = 14) Wild-type (n = 11)	T50 was significantly shorter in serum from Klotho^−/−^ mice than in serum from Klotho^+/−^ mice or wild-type mice.
Zarb et al., 2019 [8] Ossified blood vessels in primary familial brain calcification elicit a neurotoxic astrocyte response	Mice: Pdgfb^ret/ret^ (hypomorphs) (n = 10) Pdgfb^ret/wt^ (controls) (n = 10)	There was no significant difference in T50 values between Pdgfb^ret/ret^ mice and control animals.
Schantl et al., 2020 [9] Inhibition of vascular calcification by inositol phosphates derivatized with ethylene glycol oligomers	Rats (adenine and high-phosphate-diet): Vehicle (n = 11) (OEG_2_)_2_-IP4 5 mg/kg/d (n = 12) (OEG_2_)_2_-IP4 15 mg/kg/d (n = 11) (OEG_2_)_2_-IP4 50 mg/kg/d (n = 11)	T50 did not differ when comparing the treatment groups.
Moor et al., 2020 [10] Elevated serum magnesium lowers calcification propensity in Memo1-deficient mice	Mice: Whole-body Memo1 conditional KO (cKO) Controls	Memo1 cKO mice had no soft tissue calcifications and had a lower serum calcification propensity (i.e., a longer T50) and a higher serum magnesium concentration, relative to controls.
Ishida et al., 2021 [11] Assessment of calciprotein particle formation by AUC of the absorbance change: effect of FYB-931, a novel bisphosphonate compound	Rats (treated or not with vitamin D3 (Subcutaneous)): FYB-931 (a novel bisphosphonate) given intravenously thrice weekly for 2 weeks Etidronate given intravenously thrice weekly for 2 weeks	In rats not treated with vitamin D3, FYB-931 and etidronate inhibited the increase in absorbance in a dose-dependent manner, but T50 was prolonged in a non-dose-dependent manner by FYB-931 and not prolonged by etidronate (up to 30 µmol/L). FYB-931 showed the most potent inhibitory activity against CPP formation. In vitamin D3-treated rats, T50 was prolonged in a dose-dependent manner by FYB-931 for a concentration of 0.3 or 0.6 mg/kg, but not when it was less concentrated. In contrast, etidronate did not show a significant change in T50 values. T50 was not prolonged by etidronate.

AUC: area under curve; CKD: chronic kidney disease; cKO: whole-body memo1 conditional knockout; CPP: calciprotein particles; PDGFB: platelet-derived growth factor subunit B.

**Table 2:** Summary of observational or interventional clinical studies of serum calcification propensity, using the T50 test.

Clinical Studies			
Observational Studies			
Smith et al., 2014 [12] Serum calcification propensity predicts all-cause mortality in predialysis CKD	Median follow-up = 5.3 years Prospective cohort	N = 184 CKD stage 3–4 patients	Greater serum calcification propensity was independently associated with progressive aortic stiffening and an increased risk of all-cause mortality.
De Seigneux et al., 2015 [13] Living kidney donation does not adversely affect serum calcification propensity and markers of vascular stiffness	1-year follow-up Prospective cohort	N = 21 Living kidney donors	Kidney donation did not worsen calcification propensity or markers of the progression of vascular stiffness measured 1 year after donation.
Keyzer et al., 2016 [14] Calcification propensity and survival among renal transplant recipients	Median follow-up = 3.1 years Longitudinal cohort	N = 699 Kidney transplant recipients	A short serum T50 was associated with an increased risk of all-cause mortality, cardiovascular mortality, and graft failure.
Dahle et al., 2016 [15] Serum calcification propensity is a strong and independent determinant of cardiac and all-cause mortality in kidney transplant recipients	Median follow-up = 5.1 years Prospective cohort	N = 1435 Kidney transplant recipients	Serum T50 was strongly associated with all-cause and cardiac mortality.
Dekker et al., 2016 [16] High-flux hemodialysis and high-volume hemodiafiltration improve serum calcification propensity	Cross-sectional study	N = 64 Patients on hemodialysis (n = 30) Patients on hemodiafiltration (n = 34)	T50 increased to a similar extent in both the hemodialysis and hemodiafiltration groups.
Berchtold et al., 2016 [17] Phosphocalcic markers and calcification propensity for assessment of interstitial fibrosis and vascular lesions in kidney allograft recipients	Retrospective study	N = 129 Kidney transplant recipients	T50 and vitamin D were inversely associated with greater interstitial fibrosis severity, while PTH elevation was positively associated with greater interstitial fibrosis severity. T50 decreased as the extent of arterial lesions increased.
Pasch et al., 2017 [18] Blood calcification propensity, cardiovascular events, and survival in patients receiving hemodialysis in the EVOLVE Trial	64 months Post-hoc analysis of an interventional trial	N = 2785 Patients on hemodialysis with secondary hyperparathyroidism	A lower T50 was associated with all-cause mortality, myocardial infarction, and peripheral vascular events.
Pruijm et al., 2017 [19] Serum calcification propensity is associated with renal tissue oxygenation and resistive index in patients with arterial hypertension or chronic kidney disease	Cross-sectional study	N = 145 CKD patients (n = 58) Patients with arterial hypertension and preserved kidney function (n = 48) Healthy controls (n = 39)	Calcification propensity was higher in CKD patients and in hypertensive patients with preserved kidney function. Higher calcification propensity was associated with lower renal tissue oxygenation and perfusion, and higher renal vascular resistance and stiffness in both hypertensive patients with preserved kidney function and in CKD patients.
Lorenz et al., 2017 [20] Worsening calcification propensity precedes all-cause and cardiovascular mortality in hemodialyzed patients	24 months Prospective cohort	N = 188 Hemodialysis	T50′s rate of decline was a significant predictor of all-cause and cardiovascular mortality, while cross-sectional T50 at inclusion and 24 months were not.
Bielesz et al., 2017 [21] Calcification propensity of serum is independent of excretory renal function	Cross-sectional study	N = 118 CKD stage 1 patients (n = 16) CKD stage 2 patients (n = 22) CKD stage 3a patients (n = 14) CKD stage 3b patients (n = 23) CKD stage 4 patients (n = 24) CKD stage 5 patients (n = 19)	T50 was associated with serum phosphate, magnesium, fetuin-A, albumin, bicarbonate and cross-lap levels (but not with eGFR) in multivariate adjusted models. Alterations in mineral homeostasis and serum protein composition (i.e., consequences of CKD) but not excretory kidney function per se influenced the serum calcification propensity.
Dahdal et al., 2018 [22] Serum calcification propensity is independently associated with disease activity in systemic lupus erythematosus	Cross-sectional study	N = 168 Systemic lupus erythematosus	T50 was negatively associated with systemic lupus erythematosus disease activity.
Voelkl et al., 2018 [23] Zinc inhibits phosphate-induced vascular calcification through TNFAIP3-mediated suppression of NF-κB	Cross-sectional study	N = 57 CKD patients (n = 16) Hemodialysis patients (n = 20) Controls (n = 21) N = 138 Individuals with eGFR > 60 (n = 30) CKD stage 3 patients (n = 45) CKD stage 4 patients (n = 17) CKD stage 5 patients (n = 46)	The serum zinc level and T50 tended to be lower in CKD patients than in controls. In patients on dialysis, serum zinc and T50 were lower still. Serum zinc levels were significantly correlated with T50 in controls and in patients with CKD.
Voelkl et al., 2018 [24] SGK1 induces vascular smooth muscle cell calcification through NF-κB signaling	Cross-sectional study	N = 14 Hemodialysis patients (n = 7) Healthy volunteers (n = 7)	Serum calcification propensity was significantly higher in uremic serum samples than in normal serum samples.
Bostom et al., 2018 [25] Serum calcification propensity and fetuin-a: biomarkers of cardiovascular disease in kidney transplant recipients	Median follow-up = 2.18 years A longitudinal case-cohort analysis	N = 433 Kidney transplant recipients	A lower T50 and low fetuin-A levels were associated with a greater risk of cardiovascular disease outcomes.
Chen et al., 2019 [26] Patients with advanced chronic kidney disease and vascular calcification have a large hydrodynamic radius of secondary calciprotein particles	Cross-sectional study	N = 62 CKD stage 4–5 patients without vascular calcification (n = 22) CKD stage 4–5 patients with vascular calcification (n = 23) Healthy volunteers (n = 17)	Compared with healthy volunteers, CKD patients with or without vascular calcification had a lower T50. Among the CKD patients, there was no difference in T50 according to the presence or absence of vascular calcification.
Bullen et al., 2019 [27] Correlates of T(50) and relationships with bone mineral density in community-living older men: the osteoporotic fractures in men (MrOS) study	Cross-sectional study	N = 149 Men, aged 65 or over	No significant associations between T50 and total hip or total spine bone mineral density.
Bundy et al., 2019 [28] Serum calcification propensity and coronary artery calcification among patients with CKD: The CRIC (Chronic Renal Insufficiency Cohort) Study	Mean follow-up = 3.2 years Prospective cohort	N = 1274 (baseline) N = 780 (follow-up) CKD stage 2–4 patients	At baseline, T50 was not associated with CAC prevalence but was significantly associated with greater CAC severity among participants with prevalent CAC. Among participants with follow-up data, T50 was not associated with incident CAC but was significantly associated with CAC progression (a one-standard-deviation decrement in T50 was associated with a 28% [95% confidence interval 7–53%] greater risk of CAC progression).
Henze et al., 2019 [29] Impact of C-reactive protein on osteo-/chondrogenic transdifferentiation and calcification of vascular smooth muscle cells	Cross-sectional study	N = 309 CKD patients	The serum CRP concentration was inversely correlated with T50 in patients with moderately severe CKD.
Bundy et al., 2019 [30] Serum calcification propensity and clinical events in CKD	Mean follow-up = 7.1 years Prospective cohort	N = 3404 CKD stage 2–4 patients	After adjustment for conventional cardiovascular risk factors, higher serum calcification propensity was associated with cardiovascular events, end-stage kidney disease and all-cause mortality. This association was not independent of kidney function. After adjustment for eGFR and 24-h urinary protein, these associations were no longer significant.
Van Dijk et al., 2019 [31] Serum calcification propensity in type 1 diabetes associates with mineral stress	Median follow-up = 15.3 years Prospective cohort	N = 216 Patients with T1DM	T50 was associated with serum markers of elevated mineral stress but not with the development of long-term macrovascular complications.
Thorsen et al., 2019 [32] Vitamin D as a risk factor for patient survival after kidney transplantation: a prospective observational cohort study	Median follow-up = 82 months Prospective cohort	N = 762 Kidney transplant recipients	T50 was significantly higher in the vitamin D sufficient group (25(OH)D > 50 nmol/L) than in the group of patients with vitamin D deficiency (30–50 nmol/L) or insufficiency (<30 nmol/L). A lower T50 at 10 weeks post-transplant was associated with death and graft failure.
Ponte et al., 2020 [33] Dialysis initiation improves calcification propensity	3 months Prospective cohort	N = 58 Hemodialysis patients (n = 46) Peritoneal dialysis patients (n = 12)	Dialysis initiation significantly decreased calcification propensity.
Van Dijk et al., 2020 [34] Favorable serum calcification propensity with intraperitoneal as compared with subcutaneous insulin administration in type 1 diabetes	26 weeks Prospective, observational matched case-control study	N = 181 Patients with T1DM Intraperitoneal insulin administration (n = 39) Subcutaneous insulin administration (n = 142)	Intraperitoneal insulin administration resulted in a higher T50 than subcutaneous administration.
Eelderink et al., 2020 [35] Serum calcification propensity and the risk of cardiovascular and all-cause mortality in the general population: The PREVEND Study	Median follow-up = 8.3 years Prospective cohort	N = 6231 General population	T50 was inversely associated with circulating phosphate, age, eGFR, alcohol consumption, and an elevated risk of cardiovascular mortality in the general population. T50 was positively associated with the plasma magnesium level.
Nakatani et al., 2020 [6] Association between Serum Zinc and Calcification Propensity (T(50)) in Patients with Type 2 Diabetes Mellitus and In Vitro Effect of Exogenous Zinc on T(50)	Cross-sectional	N = 132 Patients with T2DM	The serum zinc level was found to be an independent factor associated positively with the serum T50 in patients with T2DM.
Kakajiwala et al., 2020 [36] Serum calcification propensity in children on chronic hemodialysis	12 weeks A prospective, single-center study	N = 9 Children on hemodialysis	Participants who had greater serum calcification propensity (i.e., a lower median T50 value) had a higher median calcium x phosphate product level, driven by higher phosphate concentrations. In a multivariate analysis, higher serum magnesium, calcium and fetuin-A concentrations were independently associated with a longer T50, and a higher serum phosphate was independently associated with a shorter T50.
Alesutan et al., 2021 [37] Circulating uromodulin inhibits vascular calcification by interfering with pro-inflammatory cytokine signaling	Cross-sectional study	N = 311 CKD patients N = 138 Individuals with eGFR > 60 (n = 30) CKD stage 3 patients (n = 45) CKD stage 4 patients (n = 17) CKD stage 5 patients (n = 46) N = 57 CKD patients (n = 16) Patients on hemodialysis (n = 20) Controls (n = 21)	In three independent cohorts of CKD patients, serum uromodulin concentrations were correlated with T50.
Chen et al., 2021 [38] Associations of Serum Calciprotein Particle Size and Transformation Time With Arterial Calcification, Arterial Stiffness, and Mortality in Incident Hemodialysis Patients	Median follow-up = 3.5 years Prospective cohort	N = 402 Hemodialysis patients	Baseline T50 and CPP2 size were not associated with the presence and severity of coronary arterial calcification and thoracic aortic calcification, or repeated measures of pulse wave velocity. There was no association between baseline T50 and the risk of death. The size of the CPP aggregates was negatively correlated with T50.
Mencke et al., 2021 [39] Serum calcification propensity is associated with HbA1c in type 2 diabetes mellitus	Cross-sectional study	N = 932 Patients with T2DM	Serum calcification propensity was negatively and independently associated with the HbA1c level. T50 was not associated with previous macrovascular events or the presence of microvascular disease.
Bojic et al., 2021 [40] Propensity for calcification in serum associates with 2-year cardiovascular mortality in ischemic heart failure with reduced ejection fraction	Median follow-up = 3.2 years Prospective cohort	N = 306 Patients with chronic heart failure and reduced ejection fraction (HfrEF)	T50 was associated with 2-year cardiovascular mortality in patients with ischemic HfrEF but not in patients with non-ischemic HfrEF. There were no significant differences in the severity and the underlying form of HfrEF (ischemic vs. non-ischemic) between T50 tertiles. There were significant differences in serum phosphate, albumin and intact FGF-23 levels between T50 tertiles.
De Haan et al., 2022 [41] Genetic determinants of serum calcification propensity and cardiovascular outcomes in the general population	Genome-wide association study + a two-sample Mendelian randomization study	N = 2739 members of the general population + N = 8566 members of the general population	Three independent genome-wide-significant single nucleotide polymorphisms in the AHSG gene (encoding fetuin-A) were identified: rs4917, rs2077119 and rs9870756 together explained 18.3% of the variance in T50. Mendelian randomization did not evidence a causal effect of T50 on cardiovascular outcomes in the general population. In patient-level analyses, rs9870756 was associated with a primary composite endpoint of all-cause mortality or cardiovascular disease and all-cause mortality alone. In patients with T2DM or CKD, the association between rs9870756 and the primary composite endpoint was stronger.
Kantauskaite et al., 2022 [42] Serum calcification propensity and calcification of the abdominal aorta in patients with primary aldosteronism	Median follow-up: Patients with primary aldosteronism (PA) = 403 days Patients with resistant hypertension (RH) = 389 days Prospective cohort	N = 94 Patients with PA (n = 66) Patients with RH (n = 28)	In patients with PA, a higher aldosterone-to-renin ratio was associated with a lower T50. The decline in T50 over the follow-up period was associated with higher calcium levels, an increase in phosphate levels, and a decrease in magnesium levels. In both the PA and RH groups, a higher atherosclerotic cardiovascular disease score was associated with a lower T50. Eighteen patients with PA underwent a CT scan of the abdomen: T50 was negatively associated with the extent of vascular calcification in the abdominal aorta.
**Interventional studies**			
Bristow et al., 2016 [43] Acute and 3-month effects of calcium carbonate on the calcification propensity of serum and regulators of vascular calcification: secondary analysis of a randomized controlled trial	3 months A randomized controlled trial	N = 41 Postmenopausal women Placebo (n = 21) Calcium carbonate 1 g/day (n = 20)	T50 declined in both groups. The changes were slightly but not significantly greater in the calcium carbonate group.
Bressendorff et al., 2016 [44] Oral magnesium supplementation in chronic kidney disease stages 3 and 4: efficacy, safety, and effect on serum calcification propensity-a prospective randomized double-blinded placebo-controlled clinical trial	8 weeks Prospective, double-blind, placebo-controlled, randomized clinical trial	N = 36 CKD stage 3–4 patients: Placebo (n = 12) Mg hydroxide 360 mg x1 (15 mmol/d elemental Mg) (n = 12) Mg hydroxide 360 mg x2 (30 mmol/d elemental Mg) (n = 12)	Oral Mg supplementation did not increase intracellular Mg levels. T50 increased significantly over baseline at weeks 4 and 8 in the Mg 30 mmol/d group. T50 increased significantly over baseline only at week 4 in the Mg 15 mmol/d group. There were no significant changes in the placebo group.
Smerud et al., 2017 [45] A rapid and sustained improvement of calcification propensity score (serum T(50)) after successful kidney transplantation: Reanalysis of a randomized controlled trial of ibandronate	1 year Post-hoc analysis of a prospective, double-blind, placebo-controlled, randomized controlled trial	N = 123 Kidney transplant recipients: Intravenous ibandronate (n = 65) Intravenous placebo (n = 58)	T50 increased from baseline to 10 weeks after transplantation, with no further change after 1 year. Ibandronate had no effect on T50, relative to placebo.
Lorenz et al., 2018 [46] Acetate-free, citrate-acidified bicarbonate dialysis improves serum calcification propensity-a preliminary study	3 months Pre-post-quasi-interventional study	N = 78 Hemodialysis patients: Citrate-acidified, standard bicarbonate dialysis solution for 3 months, then acetate-acidified, standard bicarbonate dialysis for another 3 months (n = 47) Citrate-acidified, standard bicarbonate dialysis solution for 3 months (n = 31)	Three months of dialysis with acetate-free, citrate-acidified, bicarbonate dialysis solution was associated with a longer T50, compared with acetate-acidified bicarbonate dialysis solution.
Mohammad et al., 2018 [47] A controlled increase in dietary phosphate elevates BP in healthy human subjects	11 weeks Prospective, randomized, single-blind study	N = 20 Healthy young adults: High phosphate (n = 10): regular diet + 1 mmol/kg bodyweight/d of Na as neutral sodium phosphate Low phosphate (n = 10): regular diet + lanthanum 750 mg 3x/d + 0.7 mmol/kg bodyweight/d of Na as NaCl After 6 weeks, both groups received vitamin D3 (600000 U)	Modulation of dietary phosphate loading did not significantly affect T50.
Bressendorff et al., 2018 [48] The effect of increasing dialysate magnesium on serum calcification propensity in subjects with end stage kidney disease: a randomized, controlled clinical trial	28 days Single-center, double blind, parallel group, controlled randomized clinical trial	N = 57 Hemodialysis patients: Dialysate Mg 1.0 mEq/L (n = 29) Dialysate Mg 2.0 mEq/L (n = 28)	Increasing the dialysate Mg level increased T50 in patients on maintenance hemodialysis, relative to standard-dialysate Mg. T50 returned to baseline levels when the high-dialysate Mg group was switched back to dialysate with 1.0 mEq/L Mg.
Ussif et al., 2018 [49] Paricalcitol supplementation during the first year after kidney transplantation does not affect calcification propensity score	1 year Open-label, randomized, controlled trial	N = 76 Kidney transplant recipients: Paricalcitol (n = 37) Placebo (n = 39)	Paricalcitol had no effect on T50 during the first year following transplantation.
Kendrick et al., 2018 [50] Effect of treatment of metabolic acidosis on vascular endothelial function in patients with CKD: a pilot randomized cross-over study	14 weeks Prospective, open-label, randomized crossover study	N = 18 Patients with CKD stage 3b-4 + metabolic acidosis: NaHCO3 or placebo (6 weeks) Washout (2 weeks) Placebo or NaHCO3 (6 weeks)	Oral sodium bicarbonate supplementation had no effect on T50.
Andrews et al., 2018 [51] Examining the effects of uric acid-lowering on markers vascular of calcification and CKD-MBD; A post-hoc analysis of a randomized clinical trial	12 weeks Post-hoc analysis of a double-blind, placebo-controlled, randomized clinical trial	N = 63 Patients with stage 3 CKD + hyperuricemia: Allopurinol 100 mg/d in week 1, 200 mg/d in week 2, 300 mg/d in week 3–12 (n = 29) Placebo (n = 34)	Allopurinol lowered uric acid levels but had no effect on T50 and CKD-mineral and bone disorder parameters.
Quiñones et al., 2019 [52] Control of metabolic predisposition to cardiovascular complications of chronic kidney disease by effervescent calcium magnesium citrate: a feasibility study	3 weeks Randomized crossover study	N = 18 Patients with stage 3 (n = 9) or 5D (n = 9) CKD: Calcium magnesium citrate or calcium acetate (1 week) Washout (1 week) Calcium acetate or calcium magnesium citrate (1 week)	In stage 3 CKD, neither calcium magnesium citrate nor calcium acetate altered T50. In stage 5D CKD, calcium magnesium citrate was associated with a significantly longer T50 and calcium acetate was not associated with any change.
Aigner et al., 2019 [53] Oral Sodium Bicarbonate Supplementation Does Not Affect Serum Calcification Propensity in Patients with Chronic Kidney Disease and Chronic Metabolic Acidosis	4 weeks Randomized controlled trial	N = 35 Patients with stage 3–4 CKD + chronic metabolic acidosis: Intervention group: high-dose oral sodium bicarbonate (n = 18) Rescue group: rescue therapy with sodium bicarbonate if necessary (n = 17)	Oral sodium bicarbonate supplementation had no effect on T50 in CKD patients with acidosis.
Ter Meulen et al., 2019 [54] Citric-acid dialysate improves the calcification propensity of hemodialysis patients: A multicenter prospective randomized cross-over trial	4 weeks Prospective multicenter randomized crossover study	N = 18 Hemodialysis patients: Acetate dialysate solution with a Ca concentration of 1.5 mmol/L (1 week) Acetate dialysate solution with a Ca concentration of 1.25 mmol/L or citric acid dialysate solution with a Ca concentration of 1.5 mmol/L (1 week) Acetate dialysate solution with a Ca concentration of 1.5 mmol/L (1 week) Citric acid dialysate solution with a Ca concentration of 1.5 mmol/L or acetate dialysate solution with a Ca concentration of 1.25 mmol/L (1 week)	Citric acid-buffered dialysis solution was associated with a longer T50, relative to acetate-buffered solution.
Smith et al., 2020 [55] Effect of sevelamer on calciprotein particles in hemodialysis patients: the sevelamer versus calcium to reduce fetuin-a-containing calciprotein particles in dialysis (ScaRF) randomized controlled trial	24 weeks Multicenter, 3-arm, parallel group, open-label randomized controlled trial	N = 31 Hemodialysis patients: Calcium carbonate (CC) (n = 11) Sevelamer hydrochloride (SH) (n = 11) Sevelamer carbonate(SC) (n = 9)	At 24 weeks, the serum CPP-1 level (but not the CPP-2 level), aortic pulse wave velocity and interleukin-8 levels were lower in the SH and SC groups than in the CC group. T50 increased from baseline over 24 weeks to the same extent in all three groups.
Thiem et al., 2020 [56] The effect of phosphate binder therapy with sucroferric oxyhydroxide on calcification propensity in chronic hemodialysis patients: a randomized, controlled, crossover trial	6 weeks Open-label, single-center, crossover, randomized controlled trial	N = 39 Hemodialysis + hyperphosphatemia Sucroferric oxyhydroxide 2000 mg/d or 250 mg/d (2 weeks) Washout (2 weeks) Sucroferric oxyhydroxide 250 mg/d or 2000 mg/d (2 weeks)	Treatment with the phosphate binder sucroferric oxyhydroxide significantly increased T50 and decreased serum phosphate levels.
Ketteler et al., 2020 [57] Efficacy and safety of a novel nicotinamide modified-release formulation in the treatment of refractory hyperphosphatemia in patients receiving hemodialysis-a randomized clinical trial	12 weeks Multicenter, double-blind, placebo-controlled, prospective randomized clinical trial	N = 722 Patient with hemodialysis + refractory hyperphosphatemia (i.e., despite treatment with phosphate binder): Modified-release nicotinamide + phosphate binder (n = 539) Placebo + phosphate binder (n = 183)	A combination of modified-release nicotinamide and an oral phosphate binder was associated with significantly lower serum phosphate and intact PTH levels and a longer T50, compared with a combination of placebo and phosphate binder.
Shoji et al., 2021 [58] Comparative effects of etelcalcetide and maxacalcitol on serum calcification propensity in secondary hyperparathyroidism: a randomized clinical trial	12 months Multicenter, open-label, blind end-point, randomized controlled trial	N = 321 Patients with secondary hyperparathyroidism on hemodialysis Etelcalcetide (n = 165) Maxacalcitol (n = 156)	The increase in T50 was significantly greater in the etelcalcetide group than in the maxacalcitol group.
Hammer et al., 2021 [59] Protective effects of spironolactone on vascular calcification in chronic kidney disease	40 weeks Multicenter, double-blind, placebo-controlled randomized clinical trial	N = 85 Hemodialysis patients: Spironolactone 50 mg/d (n = 45) Placebo (n = 40)	Serum calcification propensity was lower in hemodialysis patients treated with spironolactone, relative to placebo.
Wang et al., 2022 [60] Long-term effects of sevelamer on vascular calcification, arterial stiffness, and calcification propensity in patients receiving peritoneal dialysis: the randomized pilot SERENE (Sevelamer on Vascular Calcification, Arterial Stiffness) Trial	2 years Prospective, multicenter, open-label, randomized pilot study	N = 60 Peritoneal dialysis patients: Sevelamer 800 mg 3x/d (or titration up to 1200 mg 3x/d) (n = 29) Sevelamer 400 mg 3x/d + calcium carbonate	A combination of sevelamer (used as a second-line, low-dose therapy) and calcium carbonate had much the same effects on coronary artery calcium score, aortic valve calcium score, mitral annulus calcium score, pulse wave velocity, and T50 as first-line (high-dose) sevelamer therapy. After 2 years of treatment, there were no significant within-group and between-group differences in T50 or serum calcium and phosphate.
Tiong et al., 2022 [61] Effect of nutritional calcium and phosphate loading on calciprotein particle kinetics in adults with normal and impaired kidney function	240 min Controlled study	N = 30 CKD patients (n = 14) and healthy controls (n = 16): Standardized meal: Sanitarium^®^ Up&Go^®^ liquid breakfast, 250 mL (300 mg calcium, 188 mg phosphate)	In both groups, there was an early but transient within-group increase from fasting levels in T50. There were no pairwise between-group differences. There was a strong correlation between deviations from baseline in T50 and in fetuin-A.

## Data Availability

No new data were created or analyzed in this systematic review. Data sharing is not applicable to this manuscript.

## References

[1] Tian W.B., Zhang W.S., Jiang C.Q., Liu X.Y., Jin Y.L., Lam T.H., Cheng K.K., Xu L. (2022). Aortic Arch Calcification and Risk of All-Cause Mortality and Cardiovascular Disease: The Guangzhou Biobank Cohort Study. Lancet Reg. Health—West. Pac..

[2] Shanahan C.M. (2013). Mechanisms of Vascular Calcification in CKD—Evidence for Premature Ageing?. Nat. Rev. Nephrol..

[3] Liabeuf S., Delanaye P., Cavalier É., Guérin A., Kamel S., Massy Z.A., Néphrologie P. (2015). le groupe de travail « B. des calcifications vasculaires » de la S. et de la S. de Cardiovascular Calcification Inhibitors. Ann. Biol. Clin..

[4] Pasch A., Farese S., Gräber S., Wald J., Richtering W., Floege J., Jahnen-Dechent W. (2012). Nanoparticle-Based Test Measures Overall Propensity for Calcification in Serum. JASN.

[5] ter Braake A.D., Eelderink C., Zeper L.W., Pasch A., Bakker S.J.L., de Borst M.H., Hoenderop J.G.J., de Baaij J.H.F. (2020). Calciprotein Particle Inhibition Explains Magnesium-Mediated Protection against Vascular Calcification. Nephrol. Dial. Transplant..

[6] Nakatani S., Mori K., Sonoda M., Nishide K., Uedono H., Tsuda A., Emoto M., Shoji T. (2020). Association between Serum Zinc and Calcification Propensity (T50) in Patients with Type 2 Diabetes Mellitus and In Vitro Effect of Exogenous Zinc on T50. Biomedicines.

[7] Mencke R., Sijbesma J.W.A., Doorduin J., Hoenderop J.G., Pasch A., Slart R.H.J.A., Hillebrands J.L. (2018). Klotho in Vascular Biology.

[8] Zarb Y., Weber-Stadlbauer U., Kirschenbaum D., Kindler D.R., Richetto J., Keller D., Rademakers R., Dickson D.W., Pasch A., Byzova T. (2019). Ossified Blood Vessels in Primary Familial Brain Calcification Elicit a Neurotoxic Astrocyte Response. Brain.

[9] Schantl A.E., Verhulst A., Neven E., Behets G.J., D’Haese P.C., Maillard M., Mordasini D., Phan O., Burnier M., Spaggiari D. (2020). Inhibition of Vascular Calcification by Inositol Phosphates Derivatized with Ethylene Glycol Oligomers. Nat. Commun..

[10] Moor M.B., Ramakrishnan S.K., Legrand F., Bachtler M., Koesters R., Hynes N.E., Pasch A., Bonny O. (2020). Elevated Serum Magnesium Lowers Calcification Propensity in Memo1-Deficient Mice. PLoS ONE.

[11] Ishida K., Ashizawa N., Morikane S., Kurita N., Kobashi S., Iwanaga T. (2021). Assessment of Calciprotein Particle Formation by AUC of the Absorbance Change: Effect of FYB-931, a Novel Bisphosphonate Compound. J. Pharm. Pharmacol..

[12] Smith E.R., Ford M.L., Tomlinson L.A., Bodenham E., McMahon L.P., Farese S., Rajkumar C., Holt S.G., Pasch A. (2014). Serum Calcification Propensity Predicts All-Cause Mortality in Predialysis CKD. JASN.

[13] de Seigneux S., Ponte B., Berchtold L., Hadaya K., Martin P.-Y., Pasch A. (2015). Living Kidney Donation Does Not Adversely Affect Serum Calcification Propensity and Markers of Vascular Stiffness. Transpl. Int..

[14] Keyzer C.A., de Borst M.H., van den Berg E., Jahnen-Dechent W., Arampatzis S., Farese S., Bergmann I.P., Floege J., Navis G., Bakker S.J.L. (2016). Calcification Propensity and Survival among Renal Transplant Recipients. JASN.

[15] Dahle D.O., Åsberg A., Hartmann A., Holdaas H., Bachtler M., Jenssen T.G., Dionisi M., Pasch A. (2016). Serum Calcification Propensity Is a Strong and Independent Determinant of Cardiac and All-Cause Mortality in Kidney Transplant Recipients. Am. J. Transplant..

[16] Dekker M., Pasch A., van der Sande F., Konings C., Bachtler M., Dionisi M., Meier M., Kooman J., Canaud B. (2016). High-Flux Hemodialysis and High-Volume Hemodiafiltration Improve Serum Calcification Propensity. PLoS ONE.

[17] Berchtold L., Ponte B., Moll S., Hadaya K., Seyde O., Bachtler M., Vallée J.-P., Martin P.-Y., Pasch A., Seigneux S. (2016). de Phosphocalcic Markers and Calcification Propensity for Assessment of Interstitial Fibrosis and Vascular Lesions in Kidney Allograft Recipients. PLoS ONE.

[18] Pasch A., Block G.A., Bachtler M., Smith E.R., Jahnen-Dechent W., Arampatzis S., Chertow G.M., Parfrey P., Ma X., Floege J. (2017). Blood Calcification Propensity, Cardiovascular Events, and Survival in Patients Receiving Hemodialysis in the EVOLVE Trial. CJASN.

[19] Pruijm M., Lu Y., Megdiche F., Piskunowicz M., Milani B., Stuber M., Bachtler M., Vogt B., Burnier M., Pasch A. (2017). Serum Calcification Propensity Is Associated with Renal Tissue Oxygenation and Resistive Index in Patients with Arterial Hypertension or Chronic Kidney Disease. J. Hypertens..

[20] Lorenz G., Steubl D., Kemmner S., Pasch A., Koch-Sembdner W., Pham D., Haller B., Bachmann Q., Mayer C.C., Wassertheurer S. (2017). Worsening Calcification Propensity Precedes All-Cause and Cardiovascular Mortality in Haemodialyzed Patients. Sci. Rep..

[21] Bielesz B., Reiter T., Marculescu R., Gleiss A., Bojic M., Kieweg H., Cejka D. (2017). Calcification Propensity of Serum Is Independent of Excretory Renal Function. Sci. Rep..

[22] Dahdal S., Devetzis V., Chalikias G., Tziakas D., Chizzolini C., Ribi C., Trendelenburg M., Eisenberger U., Hauser T., Pasch A. (2018). Serum Calcification Propensity Is Independently Associated with Disease Activity in Systemic Lupus Erythematosus. PLoS ONE.

[23] Voelkl J., Tuffaha R., Luong T.T.D., Zickler D., Masyout J., Feger M., Verheyen N., Blaschke F., Kuro-o M., Tomaschitz A. (2018). Zinc Inhibits Phosphate-Induced Vascular Calcification through TNFAIP3-Mediated Suppression of NF-ΚB. JASN.

[24] Voelkl J., Luong T.T.D., Tuffaha R., Musculus K., Auer T., Lian X., Daniel C., Zickler D., Boehme B., Sacherer M. (2018). SGK1 Induces Vascular Smooth Muscle Cell Calcification through NF-κB Signaling. J. Clin. Investig..

[25] Bostom A., Pasch A., Madsen T., Roberts M.B., Franceschini N., Steubl D., Garimella P.S., Ix J.H., Tuttle K.R., Ivanova A. (2018). Serum Calcification Propensity and Fetuin-A: Biomarkers of Cardiovascular Disease in Kidney Transplant Recipients. AJN.

[26] Chen W., Anokhina V., Dieudonne G., Abramowitz M.K., Kashyap R., Yan C., Wu T.T., de Mesy Bentley K.L., Miller B.L., Bushinsky D.A. (2019). Patients with Advanced Chronic Kidney Disease and Vascular Calcification Have a Large Hydrodynamic Radius of Secondary Calciprotein Particles. Nephrol. Dial. Transplant..

[27] Bullen A.L., Anderson C.A.M., Hooker E.R., Kado D.M., Orwoll E., Pasch A., Ix J.H. (2019). Correlates of T50 and Relationships with Bone Mineral Density in Community-Living Older Men: The Osteoporotic Fractures in Men (MrOS) Study. Osteoporos. Int..

[28] Bundy J.D., Cai X., Scialla J.J., Dobre M.A., Chen J., Hsu C., Leonard M.B., Go A.S., Rao P.S., Lash J.P. (2019). Serum Calcification Propensity and Coronary Artery Calcification Among Patients With CKD: The CRIC (Chronic Renal Insufficiency Cohort) Study. Am. J. Kidney Dis..

[29] Henze L.A., Luong T.T.D., Boehme B., Masyout J., Schneider M.P., Brachs S., Lang F., Pieske B., Pasch A., Eckardt K.-U. (2019). Impact of C-Reactive Protein on Osteo-/Chondrogenic Transdifferentiation and Calcification of Vascular Smooth Muscle Cells. Aging.

[30] Bundy J.D., Cai X., Mehta R.C., Scialla J.J., de Boer I.H., Hsu C., Go A.S., Dobre M.A., Chen J., Rao P.S. (2019). Serum Calcification Propensity and Clinical Events in CKD. CJASN.

[31] van Dijk P.R., Hop H., Waanders F., Mulder U.J., Pasch A., Hillebrands J.-L., van Goor H., Bilo H.J.G. (2019). Serum Calcification Propensity in Type 1 Diabetes Associates with Mineral Stress. Diabetes Res. Clin. Pract..

[32] Thorsen I.S., Bleskestad I.H., Åsberg A., Hartmann A., Skadberg Ø., Brede C., Ueland T., Pasch A., Reisæter A.V., Gøransson L.G. (2019). Vitamin D as a Risk Factor for Patient Survival after Kidney Transplantation: A Prospective Observational Cohort Study. Clin. Transplant..

[33] Ponte B., Pruijm M., Pasch A., Dufey-Teso A., Martin P.-Y., de Seigneux S. (2020). Dialysis Initiation Improves Calcification Propensity. Nephrol. Dial. Transplant..

[34] van Dijk P.R., Waanders F., Pasch A., Logtenberg S.J.J., Vriesendorp T., Groenier K.H., Hillebrands J.-L., Kleefstra N., Gans R.O.B., van Goor H. (2020). Favourable Serum Calcification Propensity with Intraperitoneal as Compared with Subcutaneous Insulin Administration in Type 1 Diabetes. Ther. Adv. Endocrinol..

[35] Eelderink C., te Velde-Keyzer C.A., Frenay A.-R.S., Vermeulen E.A., Bachtler M., Aghagolzadeh P., van Dijk P.R., Gansevoort R.T., Vervloet M.G., Hillebrands J.-L. (2020). Serum Calcification Propensity and the Risk of Cardiovascular and All-Cause Mortality in the General Population. Arterioscler. Thromb. Vasc. Biol..

[36] Kakajiwala A., Pasch A., Rogers R., Hoofnagle A., Meloni S., Furth S.L., Leonard M.B., Copelovitch L., Denburg M.R. (2020). Serum Calcification Propensity in Children on Chronic Hemodialysis. Kidney Int. Rep..

[37] Alesutan I., Luong T.T.D., Schelski N., Masyout J., Hille S., Schneider M.P., Graham D., Zickler D., Verheyen N., Estepa M. (2021). Circulating Uromodulin Inhibits Vascular Calcification by Interfering with Pro-Inflammatory Cytokine Signalling. Cardiovasc. Res..

[38] Chen W., Fitzpatrick J., Monroy-Trujillo J.M., Sozio S.M., Jaar B.G., Estrella M.M., Serrano J., Anokhina V., Miller B.L., Melamed M.L. (2021). Associations of Serum Calciprotein Particle Size and Transformation Time With Arterial Calcification, Arterial Stiffness, and Mortality in Incident Hemodialysis Patients. Am. J. Kidney Dis..

[39] Mencke R., van der Vaart A., Pasch A., Harms G., Waanders F., Bilo H.J.G., van Goor H., Hillebrands J.-L., Dijk P.R. (2021). van Serum Calcification Propensity Is Associated with HbA1c in Type 2 Diabetes Mellitus. BMJ Open Diabetes Res. Care.

[40] Bojic M., Koller L., Cejka D., Niessner A., Bielesz B. (2021). Propensity for Calcification in Serum Associates With 2-Year Cardiovascular Mortality in Ischemic Heart Failure With Reduced Ejection Fraction. Front. Med..

[41] de Haan A., Ahmadizar F., van der Most P.J., Thio C.H.L., Kamali Z., Ani A., Ghanbari M., Chaker L., van Meurs J., Ikram M.K. (2022). Genetic Determinants of Serum Calcification Propensity and Cardiovascular Outcomes in the General Population. Front. Cardiovasc. Med..

[42] Kantauskaite M., Bolten K., Boschheidgen M., Schmidt C., Kolb T., Eckardt K.U., Pasch A., Schimmöller L., Rump L.C., Voelkl J. (2022). Serum Calcification Propensity and Calcification of the Abdominal Aorta in Patients With Primary Aldosteronism. Front. Cardiovasc. Med..

[43] Bristow S.M., Gamble G.D., Pasch A., O’Neill W.C., Stewart A., Horne A.M., Reid I.R. (2016). Acute and 3-Month Effects of Calcium Carbonate on the Calcification Propensity of Serum and Regulators of Vascular Calcification: Secondary Analysis of a Randomized Controlled Trial. Osteoporos. Int..

[44] Bressendorff I., Hansen D., Schou M., Silver B., Pasch A., Bouchelouche P., Pedersen L., Rasmussen L.M., Brandi L. (2017). Oral Magnesium Supplementation in Chronic Kidney Disease Stages 3 and 4: Efficacy, Safety, and Effect on Serum Calcification Propensity—A Prospective Randomized Double-Blinded Placebo-Controlled Clinical Trial. Kidney Int. Rep..

[45] Smerud K.T., Åsberg A., Kile H., Pasch A., Dahle D.O., Bollerslev J., Godang K., Hartmann A. (2017). A Rapid and Sustained Improvement of Calcification Propensity Score (Serum T50) after Successful Kidney Transplantation: Reanalysis of a Randomized Controlled Trial of Ibandronate. Clin. Transplant..

[46] Lorenz G., Mayer C.C., Bachmann Q., Stryeck S., Braunisch M.C., Haller B., Carbajo-Lozoya J., Schmidt A., Witthauer S., Abuzahu J. (2018). Acetate-Free, Citrate-Acidified Bicarbonate Dialysis Improves Serum Calcification Propensity—A Preliminary Study. Nephrol. Dial. Transplant..

[47] Mohammad J., Scanni R., Bestmann L., Hulter H.N., Krapf R. (2018). A Controlled Increase in Dietary Phosphate Elevates BP in Healthy Human Subjects. JASN.

[48] Bressendorff I., Hansen D., Schou M., Pasch A., Brandi L. (2018). The Effect of Increasing Dialysate Magnesium on Serum Calcification Propensity in Subjects with End Stage Kidney Disease: A Randomized, Controlled Clinical Trial. CJASN.

[49] Ussif A., Pihlstrøm H., Pasch A., Holdaas H., Hartmann A., Smerud K., Åsberg A. (2018). Paricalcitol Supplementation during the First Year after Kidney Transplantation Does Not Affect Calcification Propensity Score. BMC Nephrol..

[50] Kendrick J., Shah P., Andrews E., You Z., Nowak K., Pasch A., Chonchol M. (2018). Effect of Treatment of Metabolic Acidosis on Vascular Endothelial Function in Patients with CKD: A Pilot Randomized Cross-Over Study. CJASN.

[51] Andrews E.S., Perrenoud L., Nowak K.L., You Z., Pasch A., Chonchol M., Kendrick J., Jalal D. (2018). Examining the Effects of Uric Acid-Lowering on Markers Vascular of Calcification and CKD-MBD; A Post-Hoc Analysis of a Randomized Clinical Trial. PLoS ONE.

[52] Quiñones H., Hamdi T., Sakhaee K., Pasch A., Moe O.W., Pak C.Y.C. (2019). Control of Metabolic Predisposition to Cardiovascular Complications of Chronic Kidney Disease by Effervescent Calcium Magnesium Citrate: A Feasibility Study. J. Nephrol..

[53] Aigner C., Cejka D., Sliber C., Fraunschiel M., Sunder-Plassmann G., Gaggl M. (2019). Oral Sodium Bicarbonate Supplementation Does Not Affect Serum Calcification Propensity in Patients with Chronic Kidney Disease and Chronic Metabolic Acidosis. KBR.

[54] ter Meulen K.J., Dekker M.J.E., Pasch A., Broers N.J.H., van der Sande F.M., van der Net J.B., Konings C.J.A.M., Gsponer I.M., Bachtler M.D.N., Gauly A. (2019). Citric-Acid Dialysate Improves the Calcification Propensity of Hemodialysis Patients: A Multicenter Prospective Randomized Cross-over Trial. PLoS ONE.

[55] Smith E.R., Pan F.F.M., Hewitson T.D., Toussaint N.D., Holt S.G. (2020). Effect of Sevelamer on Calciprotein Particles in Hemodialysis Patients: The Sevelamer Versus Calcium to Reduce Fetuin-A-Containing Calciprotein Particles in Dialysis (SCaRF) Randomized Controlled Trial. Kidney Int. Rep..

[56] Thiem U., Soellradl I., Robl B., Watorek E., Blum S., Dumfarth A., Marculescu R., Pasch A., Haller M.C., Cejka D. (2021). The Effect of Phosphate Binder Therapy with Sucroferric Oxyhydroxide on Calcification Propensity in Chronic Haemodialysis Patients: A Randomized, Controlled, Crossover Trial. Clin. Kidney J..

[57] Ketteler M., Wiecek A., Rosenkranz A.R., Pasch A., Rekowski J., Hellmann B., Karus M., Ammer R. (2021). Efficacy and Safety of a Novel Nicotinamide Modified-Release Formulation in the Treatment of Refractory Hyperphosphatemia in Patients Receiving Hemodialysis—A Randomized Clinical Trial. Kidney Int. Rep..

[58] Shoji T., Nakatani S., Kabata D., Mori K., Shintani A., Yoshida H., Takahashi K., Ota K., Fujii H., Ueda S. (2021). Comparative Effects of Etelcalcetide and Maxacalcitol on Serum Calcification Propensity in Secondary Hyperparathyroidism: A Randomized Clinical Trial. CJASN.

[59] Hammer F., Buehling S.S., Masyout J., Malzahn U., Hauser T., Auer T., Grebe S., Feger M., Tuffaha R., Degenhart G. (2021). Protective Effects of Spironolactone on Vascular Calcification in Chronic Kidney Disease. Biochem. Biophys. Res. Commun..

[60] Wang A.Y.-M., Pasch A., Wong C.-K., Chu I.M.-T., Tang T.-K., Chu J., Cheuk-Ying Fong C., Yau Y.-Y., Lo W.-K. (2022). Long-Term Effects of Sevelamer on Vascular Calcification, Arterial Stiffness, and Calcification Propensity in Patients Receiving Peritoneal Dialysis: The Randomized Pilot SERENE (Sevelamer on Vascular Calcification, Arterial Stiffness) Trial. Kidney Med..

[61] Tiong M.K., Cai M.M.X., Toussaint N.D., Tan S.-J., Pasch A., Smith E.R. (2022). Effect of Nutritional Calcium and Phosphate Loading on Calciprotein Particle Kinetics in Adults with Normal and Impaired Kidney Function. Sci. Rep..

